# Acute caffeine intake improves muscular strength, power, and endurance performance, reversing the time-of-day effect regardless of muscle activation level in resistance-trained males: a randomized controlled trial

**DOI:** 10.1007/s00421-025-05820-3

**Published:** 2025-06-06

**Authors:** Juan Jesús Montalvo-Alonso, Marta del Val-Manzano, Ester Cerezo-Telléz, Carmen Ferragut, David Valadés, Javier Rodríguez-Falces, Alberto Pérez-López

**Affiliations:** 1 Departamento de Ciencias Biomédicas, Área de Educación Física y Deportiva, Facultad de Medicina y Ciencias de la Salud, Universidad de Alcalá, Ctra. Madrid-Barcelona km 33,600, 28871 Alcalá de Henares, Madrid Spain; 2 Neuromusculoskeletal Physical Therapy in Stages of Life Research Group (FINEMEV), Department of Nursing and Physical Therapy, Faculty of Medicine and Health Sciences, Universidad de Alcalá, Autovía A2, km 33.200, 28805 Alcalá de Henares, Madrid Spain; 3 Department of Electrical, Electronic and Communications Engineering, Public University of Navarra (UPNA), Instituto de Investigación Sanitaria de Navarra (IdiSNA), Navarra, Spain

**Keywords:** Dietary supplements, Caffeine, Sports performance, Resistance training, Surface electromyography

## Abstract

**Introduction:**

This study examined the effects of acute caffeine intake on muscular electrical activity during strength, power, and endurance performance tests at different times of day in bench press and back squat exercises.

**Methods:**

Thirteen resistance-trained men participated in a triple-blind, cross-over, randomized controlled trial with four conditions: (a) morning with caffeine, (b) morning with placebo, (c) evening with caffeine, and (d) evening with placebo. Trials were conducted at 9:00 AM and 6:00 PM, with participants consuming caffeine or placebo (3 mg/kg) 60 min prior. Muscular strength/power was tested at 25%, 50%, 75%, and 90% one-repetition maximum (1RM) for bench press and back squat, while muscular endurance was assessed at 65% 1RM through a set-to-failure. Surface electromyography (EMG) measured muscle electrical activity.

**Results:**

In muscular strength/power tests, caffeine increased mean velocity (V_mean_) and power output (W_mean_) in the back squat at 75% (P = 0.012–0.001, g = 3.84–2.71) and 90%1RM (P = 0.043–0.009, g = 2.77–2.46) in both morning and evening trials. At 25%1RM, caffeine counteracts morning performance decline in bench press (10–11%, P = 0.001, g = 2.62–1.68) and back squat (8–11%, P = 0.010–0.003, g = 2.22–1.64). In muscular endurance tests, caffeine increased V_mean_ and W_mean_ in bench press in the morning (11–12%, P = 0.003–0.005, g = 2.55–1.89) and back squat in both morning and evening trials (6–9%, P = 0.001–0.027, g = 2.79–1.73). EMG activity remained unchanged in all conditions.

**Conclusions:**

Acute caffeine intake enhances muscular strength/power at moderate-to-high loads (75%- 90%1RM) and endurance performance (65%1RM) in the back squat while counteracting morning declines at light-load (25%1RM) for both exercises without altering muscle electrical activity.

**Supplementary Information:**

The online version contains supplementary material available at 10.1007/s00421-025-05820-3.

## Introduction

Caffeine supplementation has become increasingly common among athletes from various sports. Research on resistance exercises shows that acute caffeine consumption has a positive effect on muscular strength and power output (Montalvo-Alonso et al. 2024; Wilk et al. 2020; Pallares et al. 2013), as well as on muscular endurance (Montalvo-Alonso et al. 2024; Grgic et al. 2018), enhancing both bar velocity displacement and power output. Some neuromuscular mechanisms, such as muscle fiber conducting velocity and motor unit recruitment (Bazzucchi et al. 2011), have been proposed to be behind the effect of caffeine. However, there is limited and conflicting research about the mechanism responsible for these ergogenic effects.

Caffeine, chemically known as 1,3,7-trimethylxanthine, is widely recognized for its performance-enhancing effects in sports and physical activities. Research indicates that consuming 3–6 mg/kg of body mass can significantly boost muscular strength and power in individuals with resistance training experience (Guest et al. 2021). For instance, studies have shown improvements in mean velocity (V_mean_) and mean power output (W_mean_) by 2.9% during bench press exercises at 30% of one-repetition maximum (1RM) (Wilk et al. 2020), and by 5.4–8.5% in both bench press and back squat exercises at 25–50% of 1RM (Pallares et al. 2013). Similar benefits were observed at higher intensities (75–90% of 1RM) in back squat exercises (Ruiz-Fernandez et al. 2023; Montalvo-Alonso et al. 2024). Furthermore, a meta-analysis by Grgic et al. (2018) revealed that doses of 5–6 mg/kg of caffeine improved muscular endurance, increasing the number of repetitions performed by 14%. Notably, even lower doses of caffeine (3 mg/kg) have been found to enhance performance, as evidenced by improvements in V_mean_ and W_mean_ during bench press and back squat exercises performed to failure at 60% 1RM (Duncan et al. 2013), 65% 1RM (Montalvo-Alonso et al. 2024), and 85%1RM (Ruiz-Fernandez et al. 2023).

The ergogenic effect of caffeine seems to occur mainly through neuromuscular mechanisms by antagonizing adenosine receptors, potentially thereby facilitating increased muscle fiber conducting velocity and motor unit recruitment (Bazzucchi et al. 2011). However, these effects may vary across different muscle groups (Trevino et al. 2015; Behrens et al. 2015), presumably eliciting a greater increase in force production in large compared to small muscle groups. In studies where the effect of caffeine was evaluated in elbow flexors, no ergogenic effect was observed (Trevino et al. 2015), while studies examining knee extensor muscles (e.g., quadriceps) noted a performance improvement independently of sex and type of contraction (Behrens et al. 2015). In fact, muscle activation during maximal voluntary contraction (MVC) seems to be lower in larger muscle groups (e.g., knee extensors) than in smaller muscle groups (e.g., ankle plantar flexors) (Trevino et al. 2015).

Additionally, the type of contraction (i.e. static vs. dynamic contractions) may also affect the ergogenic effect of caffeine. This assumption is based on the results of studies that have found a caffeine-induced enhancement of strength of the knee extensors under isometric conditions (Kalmar and Cafarelli 2006, 1999), whereas no effect of caffeine on MVC strength was evident for isokinetic contractions (Jacobson and Edwards 1991). Thus, most studies on this topic have focused on maximal isometric strength, and the impact of caffeine on neuromuscular function and skeletal muscle electrical activity during dynamic contractions was mostly assessed in isokinetic tasks, while isotonic muscle contraction was scarcely studied even though they are the most commonly used type of exercise in daily life. Moreover, most of these studies are focused on the central factors, including the central nervous system’s ability to generate and maintain motor drive, regulate motor unit recruitment and firing rates, since they influence force output and resistance to fatigue. However, a broader framework that also includes peripheral mechanisms (i.e., substrate availability, calcium mobilization, sodium potassium pump efficiency, or enhanced neuromuscular transmission in the muscle fiber) is also a crucial contributor to skeletal muscle’s ability to generate strength and power. In fact, the interplay between these central and peripheral factors determines overall strength and endurance performance, with muscle activation often limiting performance before contractile function becomes the primary constraint of performance fatigability (Enoka and Duchateau 2016). Understanding how caffeine interacts with these mechanisms is vital for elucidating the ergogenic effect of this substance on muscular strength and power production capacity.

The ergogenic effects of caffeine on muscular strength, power, and endurance performance may vary depending on the time of day (Zhang et al. 2024), potentially due to circadian rhythms influencing physiological responses (Teo et al. 2011). In particular, muscular strength and power production appear to reach their highest levels later in the day. For instance, findings by Guette et al. (2005) revealed that maximal torque production was significantly lower in the early morning hours (06:00 and 10:00 h), reaching approximately 90% of peak values, whereas strength performance in the evening (18:00 h) was closer to 99% of maximum levels. Since athletes often train or compete in the morning, there is growing interest in strategies to counteract this early-day decline in performance. Caffeine is considered a potential aid in reducing the decrease in muscular strength, power and endurance performance observed during morning hours. Research suggests that caffeine’s ability to enhance skeletal muscle activation, as measured by electromyography (EMG), could play a key role in these performance improvements. As mentioned, caffeine has been shown to increase motor unit recruitment and muscle fiber excitability (Bazzucchi et al. 2011), leading to greater force production and endurance during resistance exercises (Duncan et al. 2014; Grgic et al. 2018). These effects may be more pronounced during periods of peak alertness, such as late morning or early afternoon, when neuromuscular efficiency is naturally higher (Nicolas et al. 2005). Neuromuscular efficiency, resulting from the strength and EMG ratio, is a key component of performance fatigability used to determine contractile function and muscle activation mechanism alterations (Bigland-Ritchie et al. 1986). However, further studies are needed to clarify how time-of-day interactions and caffeine-induced changes in EMG activity collectively influence performance outcomes across different exercise modalities (Mora-Rodriguez et al. 2015).

Therefore, this study aimed firstly to evaluate the acute caffeine effect on muscular electrical activity during muscular strength, power, and endurance tests performed in the bench press and back squat exercise and secondly to analyze the influence of time of day in the acute effect of caffeine on muscular strength, power and endurance performance.

## Methods

### Participants

Thirteen male resistance-trained individuals participated in the study. The sample size calculation revealed that 12 participants were sufficient for the purpose of the study to show an effect size of 0.5 (α = 0.05; 1 − β = 0.80) (v3.1, G*power, Dusseldorf University, Germany). Participants’ baseline characteristics, physical activity and dietary habits are presented in Table 1. The participants did not have any diagnosed musculoskeletal, neurological, immunological, or cardiometabolic conditions, nor were they taking any medications, drugs, stimulants, or sports supplements throughout the trial period. Although it was not an inclusion or exclusion criterion, participants were high caffeine consumers (6.00–8.99 mg/kg/day) according to Filip et al. (2020) classification. Before joining the study, they were informed about all the procedures and any potential risks or discomfort associated with the experiments. After clarifying any questions, they provided their written informed consent. The study was conducted following the principles of the Declaration of Helsinki, received approval from the University Ethical Committee and was registered with ClinicalTrial.gov (NCT06606652).

**Table 1:** Participants’ baseline characteristics

N	13
Age (yrs)	24 ± 5
Body composition	
Body mass (kg)	74.4 ± 7.0
Fat mass (kg)	8.3 ± 3.7
Fat free mass (kg)	66.1 ± 5.5
Physical activity habits and performance variables	
Total physical activity (METs-min/week)	5835 ± 2660
Sedentary time (h/day)	6.3 ± 1.4
Training experience (yrs)	5 ± 3
Upper-body training (days/week)	3 ± 1
Lower-body training (days/week)	2 ± 1
Bench press (1RM/kg body mass)	1.29 ± 0.16
Back squat (1RM/kg body mass)	1.88 ± 0.34
Dietary habits	
Energy intake (kcal/kg/day)	32.0 ± 1.93
Protein (g/kg/day)	1.93 ± 0.78
Carbohydrate (g/kg/day)	3.13 ± 0.79
Fat (g/kg/day)	1.26 ± 0.48
Total caffeine intake (mg/kg/day)	8.6 ± 6.7

Values are presented as mean ± SD

*MET* metabolic equivalent, *1RM* one-repetition maximum

### Experimental design

The research followed a randomized, triple-blind, crossover, placebo-controlled and counterbalanced design. Participants visited the laboratory five times. During the initial visit, they completed questionnaires regarding their dietary and physical activity habits, underwent body composition measurements, and participated in a familiarization session where they were introduced to the tests to be used in the trials (Table 1). On visits two through five, participants were assigned to one of four experimental conditions: a) Morning with caffeine (MCAF), b) Morning with placebo (MPLA), c) Evening with caffeine (ECAF), or d) Evening with placebo (EPLA). The times of day selected were morning (9:00 AM) and evening (6:00 PM), according to reported habitual training sessions. Each trial session was separated by a period of 3 to 7 days. Participants’ trial sequence was randomized in blocks to ensure balanced trials, using the website www.randomized.org. To maintain blinding for participants and researchers (and data analysts), an external researcher generated the random allocation sequence, assigned an alphanumeric code for each condition, and prepared and administered the supplements (caffeine or placebo) throughout the study. This researcher remained the sole custodian of the condition codes, ensuring that neither participants nor researchers were aware of the assignments until after data analysis was completed.

### Experimental procedure

The experimental procedure is illustrated in the supplementary Fig. 1.

#### Preliminary assessments

A bioelectrical impedance device measured participants’ body composition during each visit (Tanita MC-780MA, Tanita Corporation of America Inc., IL, USA). They were instructed to maintain their regular physical activity and dietary habits throughout the study, to avoid vigorous training (e.g., habitual resistance training), caffeine, stimulants, and alcohol for 24 h before the familiarization and experimental trial session, and to replicate these behaviors 24 h before each trial. To ensure that and to evaluate habitual caffeine consumption, dietary habits were assessed through a 24-h recall method, referencing data from the Spanish Food Composition Database (BEDCA) and the CESNID Food Composition tables, while physical activity levels were gauged using the International Physical Activity Questionnaire (IPAQ).

The 1RM for bench press and back squat exercises was determined on the first visit to calculate the loads (kg) equivalent to 25%, 50%, 75% and 90% 1RM for each participant (Multipower, Technogym, Spain). The initial load was set at 20 kg and was progressively increased by 10–15 kg until the mean velocity (V_mean_) reached 0.2 m/s in the bench press and 0.4 m/s in the back squat, using a linear transducer (Encoder, Chronojump Boscosystem, Spain). Further minor adjustments (< 5 kg) were made to determine the 1RM accurately. After a 10-min rest period, participants completed a familiarization session, repeating the same tests in the same sequence as in the experimental trials.

#### Supplementation protocol

The supplementation protocol started 60 min before the trial. Participants consumed caffeine at a dosage of 3 mg/kg of body mass or a placebo consisting of 3 mg/kg of maltodextrin (HSN, Granada, Spain). The supplements were dissolved in 150 ml of tap water and flavored with a calorie-free additive to mask the supplements’ flavor/bitterness and smell (MyProtein, Northwich, UK). The beverages were served in opaque shaker bottles to ensure that participants and researchers were unaware of which supplement was being consumed.

#### Measurements

The participants started with tests for muscular strength and power. These assessments measured barbell velocity in a Smith machine (Multipower, Technogym, Spain), using a linear encoder (Encoder, Chronojump Boscosystem, Spain) to record the following variables: mean velocity (V_mean_), peak velocity (V_peak_), time to reach peak velocity (Time to V_peak_), mean power (W_mean_), peak power (W_peak_), time to reach peak power (Time to W_peak_) and rate of power development (RPD) across five incremental loads 25%, 50%, 75% and 90% 1RM, for both bench press and back squat exercises. Participants completed three attempts at 25% 1RM, two at 50% 1RM, and one at 75% and 90% 1RM. In every attempt, they were instructed to control the eccentric phase, pause for two seconds during the isometric phase, and then perform the concentric phase at maximum speed (Pallarés et al. 2014; Montalvo-Alonso et al. 2024). Three minutes of passive recovery were given between sets and exercises.

Subsequently, muscular endurance was assessed. Participants performed one set at 65% 1RM in the bench press and back squat, continuing repetitions until task failure. The exercise order and loads remained consistent across trials. As with the strength and power tests, participants controlled the eccentric phase, paused for two seconds in the isometric phase, and executed the concentric phase as quickly as possible. After each set, five minutes of passive recovery were allowed. The total number of repetitions, V_mean_, V_peak_, time to V_peak_, W_mean_, W_peak_, time to W_peak_ and RPD were recorded and averaged for each participant.

Electromyography (EMG) activity was monitored throughout the tests. Data on muscle activity were captured using a validated surface EMG system (mDurance, mDurance Solutions SL, Granada, Spain). Before electrode placement, the skin was cleansed with a cotton ball soaked in alcohol to ensure optimal contact. The EMG electrodes were positioned over the muscle bellies, aligned with the orientation of the muscle fibers, specifically targeting the *pectoralis major* and *triceps brachii* during the bench press, and the *rectus femoris* and *vastus lateralis* during the back squat. Electrode placement followed the guidelines provided by Surface ElectroMyoGraphy for the Non-Invasive Assessment of Muscles (SENIAM) standards (Hermens et al. 2000). The position of the electrodes was marked with a felt pen and the electrodes were relocated in this same position across all sessions. The sampling rate for EMG signals was set at 1024 Hz. The EMG signals were processed by full-wave rectification, filtered (10–500 Hz) and normalized. During muscle contractions, the root mean square (RMS) and the mean maximal voluntary contraction (MVC) values were recorded.

After familiarization and experimental trials, participants completed questionnaires previously described in the literature about adverse effects and the blinding process (Montalvo-Alonso et al. 2024). In brief, participants filled out a questionnaire assessing their perceptions of strength/power and endurance performance, fatigue, energy, irritability, exertion, as well as any discomfort in the head, heart, muscles, or gastrointestinal system, along with any urinary or sleep issues. Each item was rated on a 10-point scale, with 1 representing the lowest intensity and 10 indicating the highest.

### Statistical analysis

Data collected in the study were analyzed using the statistical package SPSS v29.0 (SPSS Inc., Chicago, IL, USA), and figures were generated using GraphPad Prism (v8, GraphPad Software Inc., La Jolla, CA, USA). Firstly, Shapiro-Wilks was used to test the normality of the data (P > 0.05). Muscular electrical activity, strength/power, and endurance performance were analyzed using a three-way ANOVA for repeated measures according to supplements (caffeine vs placebo), time of day (morning vs evening), and exercise type (bench press vs back squat) for each load. Before conducting the ANOVA, Mauchly’s sphericity test was applied. In cases where the assumption of sphericity was violated, the Huynh–Feldt adjustment was applied to modify the degrees of freedom. Post hoc analyses were performed using the Holm-Bonferroni correction. Additionally, the McNemar test was employed to evaluate differences in side effects following beverage consumption.

Data were expressed as means with their corresponding standard deviations (SD). Statistical significance was determined at P < 0.05. Effect sizes (ES) were calculated using partial eta squared (η_p_^2^) for repeated measures ANOVA and Hedges’ (g) for pairwise comparisons.

## Results

### Muscular strength and power test

**Fig. 1:**
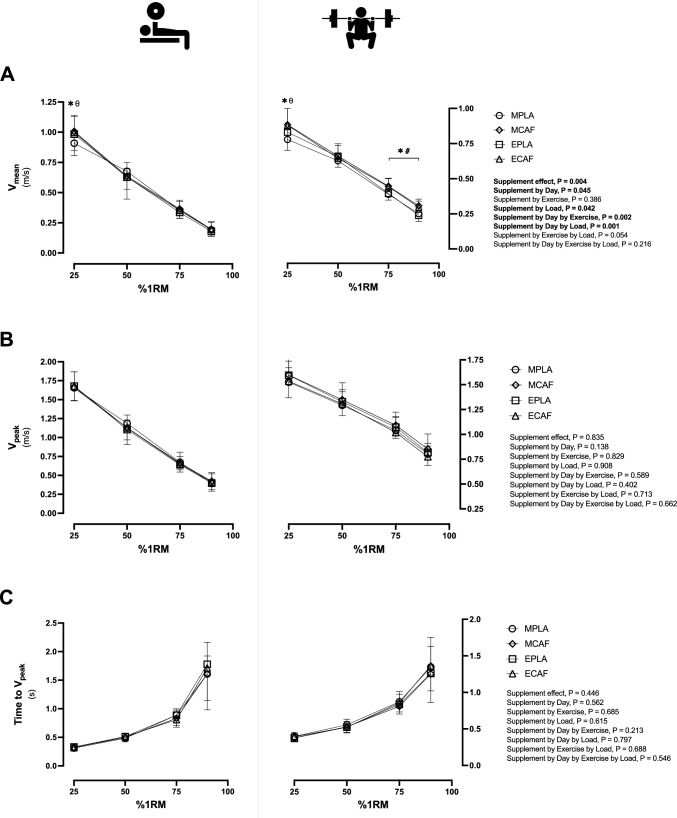
Muscular strength and power tests differences in mean, peak and time to reach peak velocity among experimental conditions. Mean velocity (V_mean_, **A**), peak velocity (V_peak_, **B**) and time to reach V_peak_ (**C**). In the three figures, bench press exercise values are illustrated in the left side, while back squat exercise values are illustrated in the right side. *MPLA* morning trial ingesting placebo, *MCAF* morning trial ingesting caffeine, *EPLA* evening trial ingesting placebo, *ECAF* evening trial ingesting caffeine. *P < 0.05 MCAF compared to MPLA; #P < 0.05 ECAF compared to EPLA; θ P < 0.05 EPLA compared to MPLA

Differences in V_mean_, V_peak_ and time to V_peak_ in bench press and back squat exercises were illustrated in Fig. 1. In V_mean_, supplement effect (P = 0.004, η_p_^2^ = 0.54), supplement by day (P = 0.045, η_p_^2^ = 0.32), supplement by load (P = 0.042, η_p_^2^ = 0.25), supplement by day by exercise (P = 0.002, η_p_^2^ = 0.58) and supplement by day by load were found (P = 0.001, η_p_^2^ = 0.48). Differences V_mean_ were found at 25%1RM in the morning when comparing caffeine to placebo in bench press (11%, P = 0.001, g = 2.62; Fig. 1A left) and back squat exercise (8%, P = 0.010, g = 2.22; Fig. 1A right), but not in the evening sessions in either bench press (1.4%, P = 0.362, g = 0.34) or back squat exercise (2%, P = 0.122, g = 0.43). Besides, at 25%1RM in the placebo condition, a higher V_mean_ was found in the evening compared to the morning trial in the bench press (8%, P = 0.002, g = 2.00) and back squat exercise (9%, P = 0.016, g = 1.64). Moreover, in back squat exercise (Fig. 1A left), caffeine intake increased performance at 75%1RM in the morning (P = 0.001, g = 3.84) and the evening trial (P = 0.012, g = 2.71), and at 90%1RM in the morning (P = 0.010, g = 2.77) and the evening trial (P = 0.009, g = 2.65). However, no other difference was found in the remaining velocity variables.

**Fig. 2:**
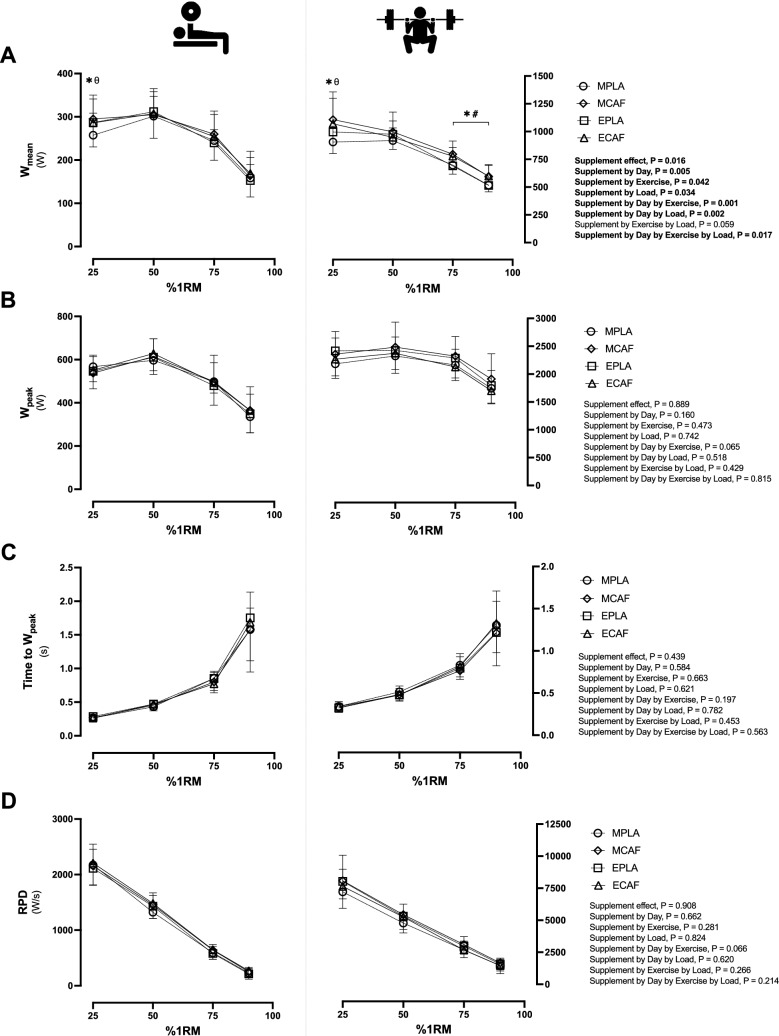
Muscular strength and power tests differences in mean, peak, time to reach peak power output and rate of power development among experimental conditions. Mean power output (Wmean, Fig. 1A), peak power (Wpeak, Fig. 2B) and time to reach Wpeak (Fig. 2C) and rate of power development (RFD, Fig. 2D). In the four figures, bench press exercise values are illustrated in the left side, while back squat exercise values are illustrated in the right side. Abbreviations: MPLA, morning trial ingesting placebo; MCAF, morning trial ingesting caffeine; EPLA, evening trial ingesting placebo; ECAF, evening trial ingesting caffeine. ^*^P 0.05 MCAF compared to MPLA; #P 0.05 ECAF compared to EPLA; θ P 0.05 EPLA compared to MPLA.

Differences in W_mean_, W_peak_, time to W_peak_ and RPD in bench press and back squat exercises were illustrated in Fig. 2. In W_mean_, supplement effect (P = 0.016, η_p_^2^ = 0.43), supplement by day (P = 0.005, η_p_^2^ = 0.52), supplement by exercise (P = 0.042, η_p_^2^ = 0.32), supplement by load (P = 0.034, η_p_^2^ = 0.23), supplement by day by exercise (P = 0.001, η_p_^2^ = 0.65), supplement by day by load (P = 0.002, η_p_^2^ = 0.45) and supplement by day by exercise by load were found (P = 0.017, η_p_^2^ = 0.35). Differences in W_mean_ were found at 25%1RM in the morning when comparing caffeine to placebo in bench press (10%, P = 0.001, g = 1.68; Fig. 2A left) and back squat exercise (11%, P = 0.003, g = 1.64; Fig. 2A right), but not in the evening sessions in either bench press (0.3%, P = 0.893, g = 0.06) or back squat exercise (1.3%, P = 0.480, g = 0.20). Besides, at 25%1RM in the placebo condition, a higher W_mean_ was found in the evening compared to the morning trial in the bench press (7%, P = 0.004, g = 2.14) and back squat exercise (8%, P = 0.005, g = 2.06). Moreover, in back squat exercise (Fig. 2A left), caffeine intake increased performance at 75%1RM in the morning (P = 0.004, g = 3.23) and the evening trial (P = 0.001, g = 3.72), and at 90%1RM in the morning (P = 0.043, g = 2.46) and the evening trial (P = 0.027, g = 2.97). However, no other difference was found in the remaining power variables.

### Muscular endurance test

**Fig. 3:**
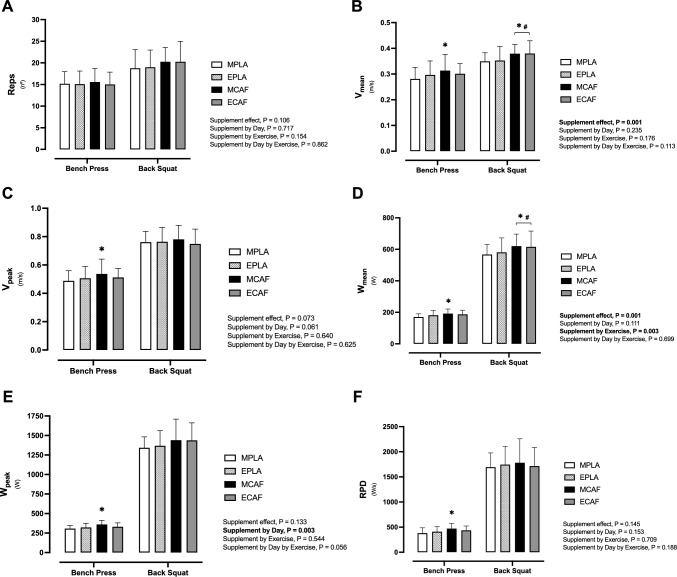
Muscular endurance test differences in the number of repetitions, mean and peak velocity and power output among experimental conditions. Number of repetitions (**A**), mean velocity (V_mean_) (**B**), peak velocity (W_peak_, **C**), mean power output (W_mean_, **D**), peak power (W_peak_, **E**) and rate of power development (RPD, **F**). *MPLA* morning trial ingesting placebo, *MCAF* morning trial ingesting caffeine, *EPLA* evening trial ingesting placebo, *ECAF* evening trial ingesting caffeine. *P < 0.05 MCAF compared to MPLA; #P < 0.05 ECAF compared to EPLA

Figure 3 illustrates differences in the number of repetitions, V_mean_, V_peak_, W_mean_, W_peak_ and RPD in the bench press and back squat exercises.

Supplement effect was found in V_mean_ (P = 0.001, η_p_^2^ = 0.76; Fig. 3B). V_mean_ increased after caffeine intake in bench press exercise during the morning (11%, P = 0.005, g = 1.89), but not in the evening trial (1.5%, P = 0.702, g = 0.34), while in the back squat exercise, the ergogenic effect of caffeine was found in the morning (8.6%, P = 0.001, g = 2.79) and the evening trial (7.9%, P = 0.002, g = 1.73).

Similarly, in W_mean_, supplement (P = 0.001, η_p_^2^ = 0.89) and supplement by exercise effect (P = 0.003, η_p_^2^ = 0.57) were found (Fig. 3D). Caffeine improved W_mean_ compared to placebo in the bench press exercise in the morning (12%, P = 0.003, g = 2.55), but not in the evening trial (3%, P = 0.445, g = 0.65). In contrast, in the back squat exercise, caffeine ergogenic effect was found in both the morning (9%, P = 0.001, g = 2.45) and evening trials (6%, P = 0.001, g = 1.15).

Finally, in W_peak_, supplement by day was found (P = 0.045, η_p_^2^ = 0.32), showing an increase in the bench press exercise in the morning trial after ingesting caffeine (11%, P = 0.027, g = 2.47; Fig. 3E).

### Muscle electrical activity

Differences in EMG obtained during the muscular strength and power and muscular endurance test were presented in Tables 2 and 3, respectively. In muscular strength and power tests performed at 25, 50, 75 and 90%1RM (Table 2), a load effect was found in RMS and MVC in *pectoralis major* (P = 0.001, η_p_^2^ = 0.73 and P = 0.001, η_p_^2^ = 0.74, respectively) and *triceps brachii* during the bench press exercise (P = 0.001, η_p_^2^ = 0.61 and P = 0.005, η_p_^2^ = 0.50, respectively), as well as in *rectus femoris* (P = 0.020, η_p_^2^ = 0.30 and P = 0.002, η_p_^2^ = 0.46) and *vastus lateralis* in the back squat exercise (P = 0.046, η_p_^2^ = 0.17 and P = 0.004, η_p_^2^ = 0.38). However, no other effect was found in any of the variables or skeletal muscles analyzed.

**Table 2:** Differences in muscle electrical activity (EMG) in muscular strength and power test among experimental conditions

			MPLA	MCAF	EPLA	ECAF	ANOVA effects (partial η2)				
			Mean ± SD	Mean ± SD	Mean ± SD	Mean ± SD	Supplement	Load	Supplement by Day	Supplement by Load	Supplement by Day by Load
25% 1RM	*Pectoralis major*	RMS (μV)	100 ± 59	85 ± 45	85 ± 36	76 ± 32	0.883 (0.004)	**0.001 (0.734)**	0.373 (0.134)	0.353 (0.162)	0.501 (0.109)
		MVC (N)	547 ± 352	452 ± 261	497 ± 154	481 ± 205	0.968 (0.001)	**0.001 (0.743)**	0.290 (0.184)	0.518 (0.106)	0.822 (0.026)
	*Triceps brachii*	RMS (μV)	97 ± 24	86 ± 31	89 ± 38	103 ± 54	0.480 (0.087)	**0.001 (0.613)**	0.210 (0.247)	0.356 (0.156)	0.749 (0.050)
		MVC (N)	535 ± 139	483 ± 175	496 ± 198	680 ± 230	0.566 (0.058)	**0.005 (0.500)**	0.182 (0.275)	0.302 (0.180)	0.412 (0.121)
	*Rectus femoris*	RMS (μV)	64 ± 31	58 ± 34	62 ± 32	63 ± 53	0.572 (0.037)	**0.020 (0.300)**	0.370 (0.090)	0.181 (0.163)	0.550 (0.066)
		MVC (N)	512 ± 284	486 ± 226	532 ± 198	487 ± 276	0.912 (0.001)	**0.002 (0.461)**	0.430 (0.071)	0.948 (0.005)	0.732 (0.026)
	*Vastus lateralis*	RMS (μV)	77 ± 36	78 ± 44	78 ± 33	76 ± 54	0.865 (0.003)	**0.046 (0.173)**	0.891 (0.002)	0.507 (0.081)	0.402 (0.096)
		MVC (N)	449 ± 153	493 ± 179	433 ± 79	418 ± 186	0.301 (0.118)	**0.004 (0.379)**	0.259 (0.139)	0.807 (0.023)	0.928 (0.004)
50% 1RM	*Pectoralis major*	RMS (μV)	120 ± 37	102 ± 53	125 ± 52	112 ± 63					
		MVC (N)	650 ± 413	557 ± 308	578 ± 180	596 ± 258					
	*Triceps brachii*	RMS (μV)	114 ± 44	92 ± 25	99 ± 33	112 ± 61					
		MVC (N)	746 ± 196	697 ± 207	657 ± 168	704 ± 194					
	*Rectus femoris*	RMS (μV)	76 ± 43	62 ± 32	77 ± 32	80 ± 50					
		MVC (N)	463 ± 269	484 ± 228	502 ± 261	470 ± 187					
	*Vastus lateralis*	RMS (μV)	82 ± 26	86 ± 59	85 ± 31	79 ± 39					
		MVC (N)	389 ± 178	443 ± 276	372 ± 77	378 ± 179					
75% 1RM	*Pectoralis major*	RMS (μV)	124 ± 102	111 ± 50	120 ± 58	116 ± 67					
		MVC (N)	671 ± 314	619 ± 328	634 ± 246	637 ± 222					
	*Triceps brachii*	RMS (μV)	123 ± 35	101 ± 45	113 ± 31	116 ± 50					
		MVC (N)	636 ± 148	640 ± 225	603 ± 125	708 ± 260					
	*Rectus femoris*	RMS (μV)	87 ± 49	71 ± 43	84 ± 53	72 ± 40					
		MVC (N)	502 ± 397	445 ± 241	549 ± 352	421 ± 194					
	*Vastus lateralis*	RMS (μV)	92 ± 44	89 ± 43	94 ± 29	90 ± 62					
		MVC (N)	495 ± 195	523 ± 202	457 ± 94	434 ± 169					
90% 1RM	*Pectoralis major*	RMS (μV)	138 ± 56	121 ± 58	141 ± 39	144 ± 113					
		MVC (N)	741 ± 449	637 ± 304	721 ± 290	704 ± 259					
	*Triceps brachii*	RMS (μV)	118 ± 47	107 ± 21	136 ± 48	143 ± 88					
		MVC (N)	801 ± 195	755 ± 166	751 ± 145	771 ± 225					
	*Rectus femoris*	RMS (μV)	96 ± 37	80 ± 37	93 ± 42	89 ± 69					
		MVC (N)	622 ± 415	574 ± 299	631 ± 308	564 ± 275					
	*Vastus lateralis*	RMS (μV)	93 ± 41	96 ± 29	91 ± 41	87 ± 49					
		MVC (N)	434 ± 167	469 ± 168	398 ± 154	394 ± 150					

Values are means ± SD

*RMS* root mean square, *MVC* maximal voluntary contraction, *MPLA* morning trial ingesting placebo, *MCAF* morning trial ingesting caffeine, *EPLA* evening trial ingesting placebo, *ECAF* evening trial ingesting caffeine

Bold values indicate* P*< 0.05

**Table 3:** Differences in muscle electrical activity (EMG) in muscular endurance test among experimental conditions

	MPLA	MCAF	EPLA	ECAF	ANOVA effects (partial η2)		
					Supplement	Day	Suplement by day
	Mean ± SD	Mean ± SD	Mean ± SD	Mean ± SD			
*Pectoralis major*							
RMS (μV)	329 ± 100	255 ± 128	322 ± 105	322 ± 105	0.479 (0.057)	0.259 (0.139)	0.259 (0.139)
MVC *(N)*	752 ± 320	630 ± 354	794 ± 272	794 ± 272	0.377 (0.087)	0.322 (0.109)	0.322 (0.109)
*Triceps brachii*							
RMS (μV)	307 ± 92	287 ± 63	297 ± 97	324 ± 112	0.463 (0.061)	0.832 (0.050)	0.330 (0.105)
MVC (N)	895 ± 295	882 ± 300	887 ± 275	998 ± 459	0.168 (0.020)	0.426 (0.072)	0.401 (0.080)
*Rectus femoris*							
RMS (μV)	207 ± 125	183 ± 127	202 ± 89	194 ± 96	0.929 (0.010)	0.397 (0.066)	0.663 (0.018)
MVC (N)	625 ± 354	659 ± 465	700 ± 309	668 ± 357	0.731 (0.011)	0.990 (< 0.001)	0.586 (0.028)
*Vastus lateralis*							
RMS (μV)	189 ± 58	191 ± 60	154 ± 66	159 ± 59	0.430 (0.058)	0.363 (0.076)	0.349 (0.080)
MVC (N)	479 ± 145	507 ± 215	400 ± 164	503 ± 210	0.382 (0.070)	0.348 (0.080)	0.350 (0.080)

Values are means ± SD

*RMS* root mean square, *MVC* maximal voluntary contraction, *MPLA* morning trial ingesting placebo, *MCAF* morning trial ingesting caffeine, *EPLA* evening trial ingesting placebo, *ECAF* evening trial ingesting caffeine

Similarly, in the muscular endurance test (Table 3), no supplement, day or supplement-by-day effect was found in EMG in any of the skeletal muscles analyzed during the bench press and back squat exercises in any of the four experimental conditions.

### Adverse effects and blinding process

No statistical differences in side effects were found in mood state, nervousness, activeness, insomnia, gastrointestinal discomfort, headache, or irritability. Finally, 38% (5 of 13) of participants correctly guessed caffeine ingestion during the morning trial, while 31% (4 of 13) correctly guessed caffeine ingestion during the evening trial.

## Discussion

The purpose of this study was to examine the effect of acute caffeine intake on muscular electrical activity during muscular strength, power, and endurance tests in the bench press and back squat exercises at different times of the day. Our results indicate that acute caffeine intake affected mean velocity and power output, improving muscular strength and power at moderate-to-high loads in back squat exercise at 75% and 90%1RM, muscular endurance performance at 65%1RM in back squat exercise, and compensated the decline in morning performance at light-load (25%1RM) in bench press and back squat exercises. However, none of these ergogenic effects were accompanied by a change in the skeletal muscle activity measured by EMG of *pectoralis major* and *triceps brachii* in bench press exercise or the *rectus femoris* and *vastus lateralis* in the back squat exercise.

In this study, acute caffeine intake caused an improvement in V_mean_ and W_mean_ of back squat exercise at 75% and 90%1RM in the morning and the evening trials, but not in the bench press exercise. Previous studies have demonstrated a similar ergogenic effect of caffeine on velocity and power output during muscular strength, power, and endurance tests. Various doses of caffeine, ranging from 3 to 9 mg/kg of body mass, have been shown to enhance velocity and power at 25%, 50%, 75%, and 90% of 1RM (Pallares et al. 2013; Mora-Rodriguez et al. 2015; Ruiz-Fernandez et al. 2023; Montalvo-Alonso et al. 2024). Pallares et al. (2013) reported that a low dose of caffeine (3 mg/kg) improved mean propulsive velocity at 25% and 50% 1RM in the bench press and full squat exercises and 75% 1RM in the full squat. Similarly, Mora-Rodriguez et al. (2015) observed this effect in the full squat but not in the bench press, while Ruiz-Fernandez et al. (2023) and Montalvo-Alonso et al. (2024) noted increases in V_mean_ and W_mean_ during the back squat but not during the bench press at moderate-to-high loads (75% and 90 of 1RM).

Similarly, in the muscular endurance test, caffeine improved V_mean_ and W_mean_ in the back squat exercise in the morning and evening trials. Previous systematic reviews and meta-analyses have demonstrated that caffeine supplementation can enhance muscular endurance (Grgic et al. 2018). This improvement is primarily attributed to an increase in the number of repetitions performed per set following the acute consumption of caffeine (Grgic et al. 2019). However, other studies have highlighted that caffeine also increases V_mean_ and W_mean_ in the bench press and back squat exercises at 60%1RM (Duncan et al. 2013), 65%1RM (Montalvo-Alonso et al. 2024) and 85%1RM (Ruiz-Fernandez et al. 2023).

Therefore, these previous results were consistent with the present observations in muscular strength, power, and endurance test, as acute caffeine consumption improved muscular strength and power performance at 75% and 90% 1RM and muscular endurance at 65%1RM by enhancing V_mean_ and W_mean_ in the back squat exercise.

The ability of caffeine to promote an improvement in muscular strength, power and endurance performance found in this study occurred regardless of skeletal muscle electrical activity. Even though RMS was significantly higher with increased loads, from 25 to 90% 1RM, no supplement effect was noted. An increase in muscle fiber conducting velocity and motor unit recruitment has been proposed as caffeine’s mechanism of action (Bazzucchi et al. 2011). When the effect of caffeine was evaluated on muscular strength, Behrens et al. (2015) reported that caffeine consumption (8 mg/kg) significantly increased peak torque for isometric, concentric, and eccentric muscle actions for the quadriceps muscle, reporting an increase in muscle activation (by EMG) in isometric and eccentric contraction. Similarly, Bazzucchi et al. (2011) found caffeine (6 mg/kg) increased the torque-angular velocity curve in maximal isokinetic contraction of elbow flexion, and effect that was accompanied by an increase in RMS and estimated muscle fiber conduction velocity obtained from EMG, suggesting that caffeine improves muscle performance during short-duration by increasing motor unit recruitment. However, some studies failed to find a different percentage of motor unit activation after caffeine ingestion (Tarnopolsky and Cupido 2000; Meyers and Cafarelli 2005); for instance, Kalmar and Cafarelli (1999) found that caffeine (6 mg/kg) increased MVC of knee extension without finding changes in H-reflex amplitude, RMS or motor unit firing rates, suggesting that caffeine increases MVC at a supraspinal level or peripheral mechanisms.

Moreover, from a motoneuron perspective, higher firing frequencies (Phillis et al. 1979), motor unit recruitment (Black et al. 2015), and occurrence of self-sustained firing in motor units were observed after caffeine consumption (Walton et al. 2002). Mackay et al. (2023) found that persistent inward currents contribution (PIC) to motoneuron firing increases with contraction intensities and is reduced after repetitive, sustained maximal contractions in *tibialis anterior*, regardless of caffeine consumption (6 mg/kg). Also, caffeine consumption attenuated neuromuscular performance reductions, allowing higher time-torque integral production during repetitive sustained maximal contractions, which was unlikely mediated by PICs (Mackay et al. 2023). Therefore, in dynamic contractions, caffeine does not seem to alter muscular strength and power via increasing motor unit firing rates or motor unit recruitment and, consequently, peripheral mechanisms inside the muscle may underpin the ergogenic effect of this substance (Rousseau et al. 1988; Fukutani et al. 2022). Evidence in dynamic exercise performing isokinetic muscle contraction supports this idea. San Juan et al. (2019) observed that caffeine (3 mg/kg) improved neuromuscular efficiency in elite boxers, but without altering EMG activity of *vastus lateralis, gluteus maximus* or *tibialis anterior* after a 30 s Wingate test. Similarly, Greer et al. (2006) found in recreationally active males that caffeine (6 mg/kg) did not cause an ergogenic effect on a Wingate test in the surface EMG records of the right *vastus lateralis* and the *gastrocnemius*. Therefore, although more studies are required to fully explore the potential influence of caffeine in dynamic exercise using isotonic contraction (e.g., resistance exercise), our study failed to detect any improvement in muscle electrical activity after the intake of low doses of caffeine (3 mg/kg) despite the increase in muscular strength and power performance caused by this supplement.

Regarding muscular endurance, some findings using single-leg knee extensor exercise support the hypothesis that caffeine might attenuate contractile function impairment (Pethick et al. 2018; Meyers and Cafarelli 2005). The rate by which evoked quadriceps twitch force declined during intermittent submaximal contractions was significantly attenuated with caffeine ingestion (Meyers and Cafarelli 2005), additionally causing an attenuation in the rate of decrease in muscle activation, measured via pre-exercise to post-exercise reduction in voluntary activation using superimposed twitch interpolation technique (Pethick et al. 2018). In whole-body exercise, Cristina-Souza et al. (2022) found a similar result in ten healthy males cycled until exhaustion; caffeine increased time to exhaustion via attenuation of exercise-induced reduction in voluntary activation and contractile function. When the effect of caffeine was evaluated on strength tasks, Morse et al. (2016) noted that the ergogenic effect of 200 mg of caffeine on the power production of single-leg knee-extensor ergometry was accompanied by a delay in the onset of the electromyographic fatigue threshold, which may indicate that caffeine may delay fatigue in the *quadriceps femoris* muscle. Also, Kalmar and Cafarelli (1999) found that 6 mg/kg of caffeine caused an increase in participants’ ability to sustain the 50% MVC, together with an increase in surface EMG and percent activation. Similarly, Duncan et al. (2014) found that 6 mg/kg of caffeine caused an increase in muscle torque production and velocity interaction of *vastus medialis* when ten participants completed six repetitions of isokinetic knee extension at three angular velocities (30º/s, 150º/s, and 300º/s); suggesting that acute caffeine ingestion improves muscle performance and increases muscle activity during short-duration maximal dynamic contractions. However, in our study, despite caffeine stimulated an improvement in V_mean_ and W_mean_ in the back squat exercise and in V_peak_, W_peak,_ and RPD in bench press of the morning trial, this ergogenic effect was not accompanied by an improvement in RMS or MVC when a set at 65%1RM was performed until task failure. Hence, this evidence may indicate that the acute effect of caffeine consumption on motor unit recruitment may be dependent on the exercise mode performed, and in the muscular endurance of dynamic isotonic contraction does not seem to play a major role, at least after 3 mg/kg of caffeine intake.

Taken together, our data revealed that 3 mg/kg of caffeine causes an increase in muscular strength, power and endurance performance at moderate-to-high loads, but this ergogenicity occurred without altering muscle electrical activity. This may support the hypothesis that the caffeine mechanism of action is attributable to peripheral factors such as the inhibition of phosphodiesterase, the stimulation of calcium ion (Ca^2+^) release from the sarcoplasmic reticulum, the increase in sodium–potassium pump activity or the antagonization benzodiazepine receptors in skeletal muscle (Davis and Green 2009; Penner et al. 1989; James et al. 2005). However, the influence of the central factor (e.g., motor unit recruitment) (Bazzucchi et al. 2011) on caffeine’s mechanism of action cannot be avoided since the changes in neural drive induced d by a low dose of caffeine (3 mg/kg) might be of such a small magnitude that it did not produce a measurable response in EMG RMS. Thus, although caffeine may modulate neuromuscular activation, the magnitude of these changes caused by low doses of caffeine is likely insufficient to be reflected in surface EMG activity.

Finally, our results also revealed that V_mean_ and W_mean_ decreased in the morning compared to the evening trial, an effect that was compensated when caffeine was ingested. This effect was found in the muscular strength and power test at 25%1RM in the bench press and back squat exercises, and in muscular endurance at 65% in the bench press but not in the back squat exercise. Caffeine has previously been proposed as an effective strategy to alleviate the decline in morning performance. Mora-Rodriguez et al. (2012) noted a range of 3.0–7.5% decrement in velocity and power output in free-weight bench press and back squat exercises performed at 75%1RM, a decrement in performance that was mitigated with acute caffeine consumption (3 mg/kg) that enhanced morning trial performance by 4.6–5.7%. Moreover, the mentioned research group (Mora-Rodriguez et al. 2015) also noted that 6 mg/kg of caffeine mitigated the decline in morning vs evening performance in muscular strength and power of bench press and back squat exercises at 25%, 50%, 75% and 90%1RM. In contrast, here, we only find a decline in morning vs evening performance at 25% 1RM in the bench press and back squat exercise, a time-of-day effect mitigated when caffeine was ingested in the morning.

The lack of time-of-day effect can be attributed to the level of training, since experienced resistance participants exhibit enhanced muscle fiber recruitment, motor unit synchronization, and neuromuscular efficiency (Santos et al. 2023), allowing for more consistent output across various conditions. However, similar 1RM normalized per kg of body mass in bench press and back squat exercise was found in the three studies: 1.15 ± 0.08 and 1.46 ± 0.15, respectively, in Mora-Rodriguez et al. (2012), ~ 1.47 and ~ 1.58, respectively, in Mora-Rodriguez et al. (2015) and to 1.29 ± 0.34 and 1.88 ± 0.34, respectively, in this study. Nonetheless, interestingly, Mora-Rodriguez et al. (Mora-Rodriguez et al. 2012) also found that the performance improvement was accompanied by an increase in isometric electrically evoked strength of the right knee and norepinephrine levels (serving as a surrogate measure of maximal muscle sympathetic nerve activation) during the morning caffeine trial compared to the morning placebo trial. A differential recruitment of motor units, by either central drive/motivation and/or motor innervation patterns, during the process of generating force by muscle may be suggested as a potential mechanism to explain time-of-day muscle strength variation (Douglas et al. 2021). Several studies have explored this idea by using electromyography to measure muscle activation during time-of-day strength measures (Gueldich et al. 2017) or by an interpolated twitch technique to override any upstream time-of-day differences with strength outcomes (Martin et al. 1999). These and other studies have reported no significant time-of-day differences in RMS during maximal voluntary isometric contractions or in maximal isometric strength in both voluntary and electrically induced muscle contractions. Here, we are aligned with these studies since we did not find differences in RMS in any skeletal muscle analyses during the bench press and back squat exercises according to time-of-day. Hence, this evidence suggests that the time-of-day variation in muscular strength and power may occur mainly peripherally in skeletal muscles rather than affecting neural structures (Sedliak et al. 2008), supporting the idea that caffeine via peripheral factors may counteract the decline in morning performance.

## Conclusions

In conclusion, low doses of caffeine (3 mg/kg) consumed acutely improve mean velocity and power output at moderate-to-high loads in muscular strength and power (75–90%1RM) and muscular endurance (65%1RM) tests in back squat exercise and compensate for the morning decrease in performance in both variables at low loads (25%1RM) in bench press and back squat exercise. However, these ergogenic effects were not accompanied by increased skeletal muscle activity measured by EMG of *pectoralis major* and *triceps brachii* in bench press exercise or the *rectus femoris* and *vastus lateralis* in the back squat exercise.

## Supplementary Information

Below is the link to the electronic supplementary material.

Supplementary file1 (PDF 277 KB) Supplementary Figure 1. Experimental procedure

## Data Availability

The dataset used and analyzed during the current study is available from the corresponding author on reasonable request.

## Abbreviations

1RM: One-repetition maximum
EMG: Electromyography
ECAF: Evening with caffeine trial
EPLA: Evening with placebo trial
MCAF: Morning with caffeine trial
MPLA: Morning with placebo trial
MVC: Maximal voluntary contraction
RMS: Root mean square
RPD: Rate of power development
V_mean_: Mean velocity
Vpeak: Peak velocity
Wmean: Mean power output
Wpeak: Peak power output
