# Supplementary material for: Acute caffeine intake improves muscular strength, power, and endurance performance, reversing the time-of-day effect regardless of muscle activation level in resistance-trained males: a randomized controlled trial
Source: Eur J Appl Physiol. 2025 Jun 6;125(11):3259–72. doi: 10.1007/s00421-025-05820-3 (PMC12528362; doi:10.1007/s00421-025-05820-3)
Supplement: Supplementary file 1 — Supplementary file1 (PDF 277 KB) Supplementary Figure 1. Experimental procedure [file 421_2025_5820_MOESM1_ESM.pdf]

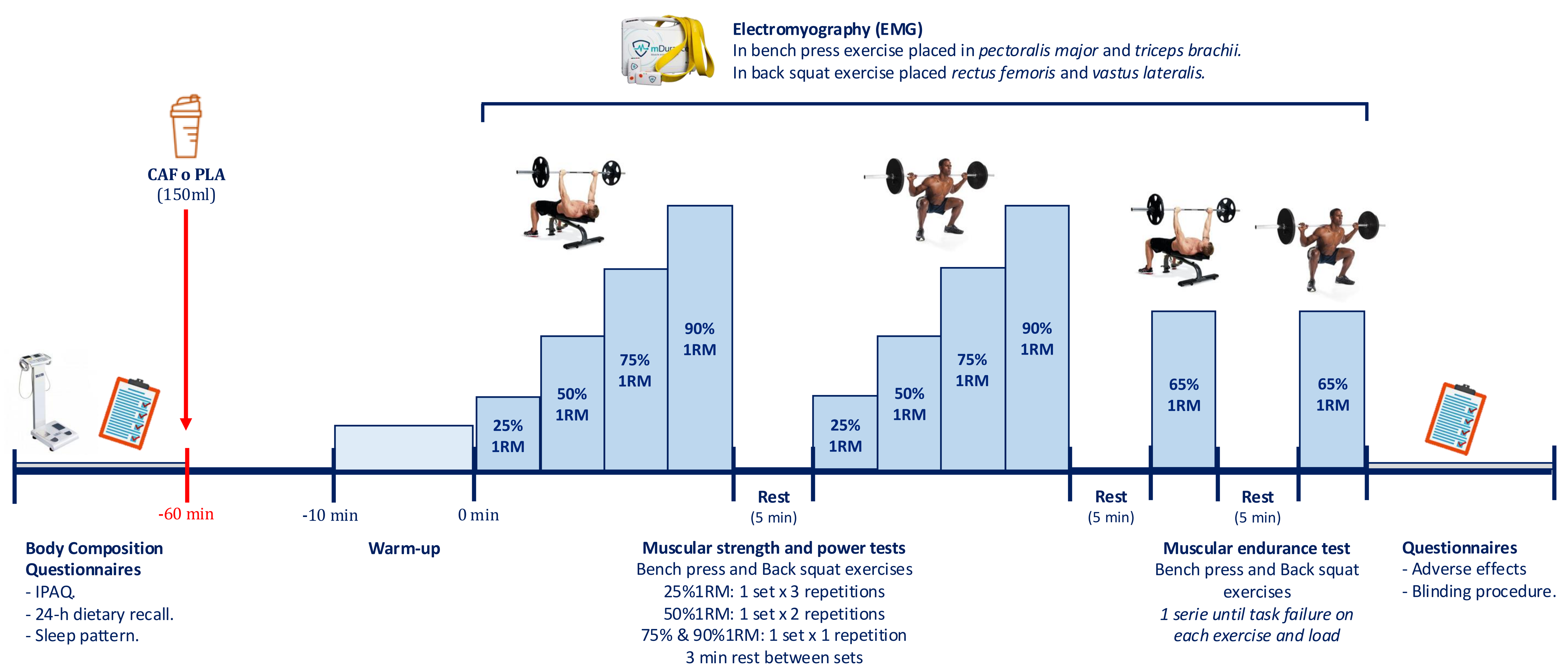
Electromyography (EMG)
In bench press exercise placed in *pectoralis major* and *triceps brachii.*
In back squat exercise placed *rectus femoris* and *vastus lateralis.*
CAF o PLA
(150ml)
25%
1RM
50%
1RM
75%
1RM
90%
1RM
25%
1RM
50%
1RM
75%
1RM
90%
1RM
65%
1RM
65%
1RM
-60 min
-10 min
0 min
Rest
(5 min)
Rest
(5 min)
Rest
(5 min)
Body Composition
Questionnaires
- IPAQ.
- 24-h dietary recall.
- Sleep pattern.
Warm-up
Muscular strength and power tests
Bench press and Back squat exercises
25%1RM: 1 set x 3 repetitions
50%1RM: 1 set x 2 repetitions
75% & 90%1RM: 1 set x 1 repetition
3 min rest between sets
Muscular endurance test
Bench press and Back squat
exercises
1 serie until task failure on
each exercise and load
Questionnaires
- Adverse effects
- Blinding procedure.
